# Association of temporary Environmental Protection Agency regulation suspension with industrial economic viability and local air quality in California, United States

**DOI:** 10.1186/s12302-021-00489-9

**Published:** 2021-04-21

**Authors:** Emily Chang, Kenneth Zhang, Margaret Paczkowski, Sara Kohler, Marco Ribeiro

**Affiliations:** 1 Math, Engineering, and Science Academy, Albemarle High School, 2775 Hydraulic Road, Charlottesville, VA 22901 USA; 2 Ancaster High School, 374 Jerseyville Road W, Ancaster, ON L9G 3K8 Canada; 3 Albemarle High School, 2775 Hydraulic Road, Charlottesville, VA 22901 USA; 4 Waseca Senior and Junior High School, 1717 2nd St NW, Waseca, MN 56093 USA; 5 Harvard University, 86 Brattle Street, Cambridge, MA 02138 USA

**Keywords:** Pollution regulations, Regulation rollback, Machine learning, Particulate matter PM_2.5_ (PM_2.5_), Employment by industry, State lockdown, Coronavirus pandemic, Oil refining sector, Manufacturing sector

## Abstract

**Background:**

This study seeks to answer two questions about the impacts of the 2020 Environmental Protection Agency’s enforcement regulation rollbacks: is this suspension bolstering the economic viability of industries as oil and manufacturing executives claim they will and are these regulations upholding the agency’s mission of protecting the environment?

**Results:**

To answer the former question, we utilized 6 months of state employment level data from California, United States, as a method of gauging the economic health of agency-regulated industries. We implemented a machine learning model to predict weekly employment data and a *t*-test to indicate any significant changes in employment. We found that, following California's state-issued stay-at-home order and the agency’s regulation suspension, oil and certain manufacturing industries had statistically significant lower employment values.

To answer the latter question, we used 10 years of PM_2.5_ levels in California, United States, as a metric for local air quality and treatment–control county pairs to isolate the impact of regulation rollbacks from the impacts of the state lockdown. Using the agency’s data, we performed a *t*-test to determine whether treatment–control county pairs experienced a significant change in PM_2.5_ levels. Even with the statewide lockdown—a measure we hypothesized would correlate with decreased mobility and pollution levels—in place, counties with oil refineries experienced the same air pollution levels when compared to historical data averaged from the years 2009 to 2019.

**Conclusions:**

In contrast to the expectation that the suspension would improve the financial health of the oil and manufacturing industry, we can conclude that these industries are not witnessing economic growth with the suspension and state shutdown in place. Additionally, counties with oil refineries could be taking advantage of these rollbacks to continue emitting the same amount of PM_2.5_, in spite of state lockdowns. For these reasons, we ask international policymakers to reconsider the suspension of enforcement regulations as these actions do not fulfill their initial expectations. We recommend the creation and maintenance of pollution control and prevention programs that develop emission baselines, mandate the construction of pollution databases, and update records of pollution emissions.

**Supplementary Information:**

The online version contains supplementary material available at 10.1186/s12302-021-00489-9.

## Background

During the COVID-19 pandemic, environmental deregulation became an international phenomenon. For example, Canada’s Alberta Energy Regulator suspended many monitoring requirements for oil and gas companies [11]. These changes were requested by the oil industry so that they could “follow public health orders” but were passed without public consultation or reasons why the changes were needed for public health reasons [27]. After 6 years of industries attempting to delay and evade meeting stringent air pollution standards, South Africa’s Minister of Environment, Forestry and Fisheries, Barbara Creecy, published air pollution standards that are twice as weak as the previous standards on the first day of South Africa’s national lockdown [3]. Other world governments that have grown laxer toward oil and gas companies include Greece which approved a controversial environmental bill that allows oil and gas exploration in previously protected areas while reducing the ability of local governments to block such investments [11].

Another notable example involves the United States of America. Since March 1, 2020, 40 rollbacks to environmental protections have been advanced or finalized, including those that affect the climate [11]. On March 26, 2020, the Environmental Protection Agency (EPA) issued guidelines stating that the agency “…does not expect to seek penalties for noncompliance…that are the result of the COVID-19 pandemic” [18] since the pandemic could constrain the ability to conduct adequate testing [4]. In doing this, the EPA effectively stated that the enforcement of air pollution regulations is temporarily suspended until further notice. Despite its lifting of pollution regulations, the EPA remains committed to protecting public health and improving air quality by reducing pollution [39], indicating that the agency does not foresee that these regulations will negatively affect environmental conditions as the suspension adheres to the agency’s mission statement. These guidelines place regulated economic sectors on an honor system, requiring industries to keep records of noncompliance but not requiring them to release these records to the public or report them to the EPA. The agency’s actions are supported by executives of top oil companies who have said that “…tighter regulations on emissions of fine soot would harm their economic viability” [13].

Additionally, certain regions in the US are more exposed to the effects of increased pollution levels, demonstrating the necessity of environmental regulations. Scholars have found that in the Appalachian region, an area exploited by industries that establish and abandon hazardous facilities, residents face severe environmental risks [36].

Most concerning are the links between pollution levels, diseases, and mortality. Before the coronavirus disease 2019 (COVID-19) outbreak, large swaths of the global population were at risk for dying from an air pollutant-related disease with the World Health Organization (WHO) estimating in 2016 that nearly 3 million people die each year from air pollution-related diseases [40]. This statistic is expected to increase as exposure to fine particulate matter (PM_2.5_), a complex mixture of small particles and liquid droplets with aerodynamic diameter ≤ 2.5 μm in the atmosphere [44], leads to a higher risk of dying from COVID-19. A Harvard study conducted by Wu et al. indicates that residents living in areas with high levels of PM_2.5_ are 8% more likely to die from COVID-19 [42], indicating that the deregulation of air pollutant emissions could intensify the growing public health crisis in the era of the COVID-19 pandemic.

Nationwide lockdowns have led to the decline of air pollutants during the COVID-19 pandemic. According to 2020 satellite data, major cities in Europe have witnessed significant reductions in nitrogen dioxide concentrations throughout middle to late March [21]. Similar results could be found in China, which experienced a 10–30% decrease in nitrogen dioxide concentrations [32].

While nationwide quarantines appear to limit pollutant emissions, pollutant emissions have been making a comeback. Analysts have witnessed that while fossil fuel consumption may have bottomed out earlier in the year, coal consumption returned to normal levels by March 30, 2020. On the same note, nitrogen dioxide levels have also returned to normal levels with provincial governments approving a surge of new coal power plant projects, indicating that pollution levels are bound to rise as industries manufacture more to make up for lost time and thus, release even more pollutants [31]. Given the current conditions of this pandemic era, the lack of regulations that could limit the emissions of pollutants ultimately puts the health of our environment and public health at risk.

Other research indicates that this downward trend in pollution levels is neither sustainable nor consistent worldwide. On one hand, a study published in the *Proceeding of the National Academy of Sciences* found that average global air quality during lockdowns improved relative to the same periods in 2019 [44]. On the other hand, a Chinese study reported “extreme particulate matter levels in northern China” [29]. This occurrence was attributed to atmospheric chemical reactions that cause particulate matter to have a nonlinear relationship with certain pollutants, such as NO_2_ and O_3_ [29]. This occurrence is not an anomaly as levels of particulate matter in Los Angeles were similar to those of previous years while primary emissions of nitrogen dioxide fell [5]. After studying data released by the EPA, another study indicated that no individual US state had lower than expected PM_2.5_ and ozone for all weeks post-COVID response [5].

The EPA claims the new guidelines will lead to increased economic viability for the manufacturing and oil sectors and will continue to uplift the EPA’s mission of protecting environmental health. However, there remains a paucity of information regarding the impact the EPA regulation suspension might have on air pollution levels and the economic viability of industries. However, a study by BW Research indicates that fossil fuel employment levels—an indicator of the economic health of the industry—have been declining ever since the COVID-19 induced economic downturn, with the oil industry losing the most workers [26]. Additionally, a study conducted by the Institute of Labor Economics researched the EPA’s March 26, 2020, enforcement suspensions and concluded that counties with pollution monitoring sites saw increases in pollution following the EPA’s rollback of enforcement [35]. If the EPA’s justifications prove to be invalid, heightened levels of air pollution could strain our nation’s healthcare system with increased COVID-19 cases without promoting the economic recovery some industries expect. We aim to analyze whether the EPA’s claims are indeed valid and draw a thorough analysis to help federal policymakers at the agency devise effective air pollution control programs so that these detrimental scenarios might be prevented.

## Methods

### Usage of economic data

Initially, to detail the circumstances of the economic shutdown created from statewide stay-at-home orders, we looked into gross state product (GSP) [2], a common measure of the size of the economy and growth rate. However, GSP is measured over the course of 3 months, a frequency that was too low. Consequently, we turned to economic proxies that were measured on a more frequent basis, including national oil consumption data [16], state oil production data [8], federal unemployment levels [41], state unemployment levels [17], and state employment levels by industry [17]. We focused on the state of California since it has the largest population and rich data.

To determine whether the rollbacks were associated with increased economic viability for economic sectors, we collected employment data on several treatment industries, or industries that we assumed the EPA regulations could impact according to the agency’s page on regulatory and compliance assistance by sector [20]. Here, employment serves as a proxy for economic health for an industry as Okun’s law states that unemployment is inversely correlated with Gross Domestic Product (GDP), an indicator of economic health [22]. Employment values from the treatment industries: Oil & Gas Extraction; Mining, except Oil & Gas; Paper Manufacturing; Wood Product Manufacturing; Construction; and Automotive Parts, were collected from the California Employment Department [17]. Using unemployment data provided by the United States Department of Labor [41], we subtracted continued unemployment claims from covered employment data to determine the total employed persons within the state of California. To compute our weekly employment values for control industries, we summed the employment values from the treatment industries and subtracted them from the total employed persons.

### Predicting NaN values using K-Nearest Neighbors imputer

We only had access to monthly local unemployment data, but needed weekly recordings to conduct an effective statistical analysis. To predict weekly unemployment values, machine learning engineers must attempt to eliminate as many noise-causing factors such as Not a Number (NaN) values, or missing values. To eliminate these factors, we needed to find an algorithm that would ignore the NaN values or substitute accurate predictions of the NaN values to provide a sufficient amount of values.

To substitute the NaN positions with accurate predictions of weekly employment values, we used different algorithms to predict those values dependent on the shape and form of the data. The monthly data that were provided was filled with data points at different checkpoints meaning that the data could be predicted by taking samples of the data. The KNNImputer algorithm was the best for filling in these values as it takes samples of missing values and the NaN values are imputed using the mean values from the nearest neighbors found in the training set. From this, we can insert the imputed values into the NaN positions, respectively, predicting future unemployment values.

### Predicting future employment values for each industry

The employment by industry data provided by the California Employment Department [17] was sparse and insufficient for predicting future values. Due to the paucity of data points provided, we had to determine an algorithm to predict future values. We initially believed that linear modeling algorithms would fit the data best due to the ability of linear algorithms to predict scarce data. Specifically, the ability of the Bayesian Ridge Regression (BRR) algorithm was favored as its natural mechanism's ability to survive in insufficient or abysmally distributed data. However, the algorithm is optimized more in some of the industry data samples than others. Due to this randomized nature, we would have had to use different algorithms to predict future values of industry data samples, introducing a level of bias in the prediction of future values based on data samples of different industries. We had to find an algorithm that was mathematically flexible enough, but could also be altered depending on the sparseness of the data to best fit the data sample of a specific treatment industry.

To eradicate bias from the models, we needed to find a regression analysis algorithm that could model the relationship between the ‘x’ and ‘y’ using the powers of a function. Polynomials are best suited for this scenario, as they are expressions that contain indeterminates and coefficients that can be adjusted to fit data samples the best and produce the most accurate predictions. To do this, we selected polynomial regression analysis, a configuration of regression analysis that is designed to model the relationship between ‘x’ and ‘y’ variables to the “nth” degree of a polynomial. This allows the function to adjust its coefficients to precisely fit the sample of data for a specific treatment industry. Computationally, we utilized the NumPy Python 3.7.7 Package to fit these polynomial functions to efficiently and sufficiently model the data.

To achieve precise modeling of the polynomial regression line that would later be fitted with the treatment industry's data, we used the Python programming language to build models based on the data that were inputted into the ‘x’ and ‘y’ variables. We proceeded to initialize two variables: one that would represent the range of the ‘x’ variables, and another variable that would represent the power of variables. The polynomial regression line was manipulated to allow us to control the power and smoothness of the polynomial regression line. Manipulative modifications did not affect the results.

### Managing air quality data

To assess local air quality, the pollutant analyzed was PM_2.5_ as it fluctuates and can quickly react to changes in the local environment [24, 25]. Data from 2009 to 2020 were collected from the EPA’s Outdoor Air Quality Index (AQI) [19]. Data were filtered to contain only measurements from California as some of the state’s counties were known for having high levels of long- and short-term particle and ozone pollution before COVID-19 [1]. Parts of the study utilized daily mean PM_2.5_ concentration levels for specific counties while others averaged these values weekly.

### Selecting treatment–control county pairs

**Fig. 1:**
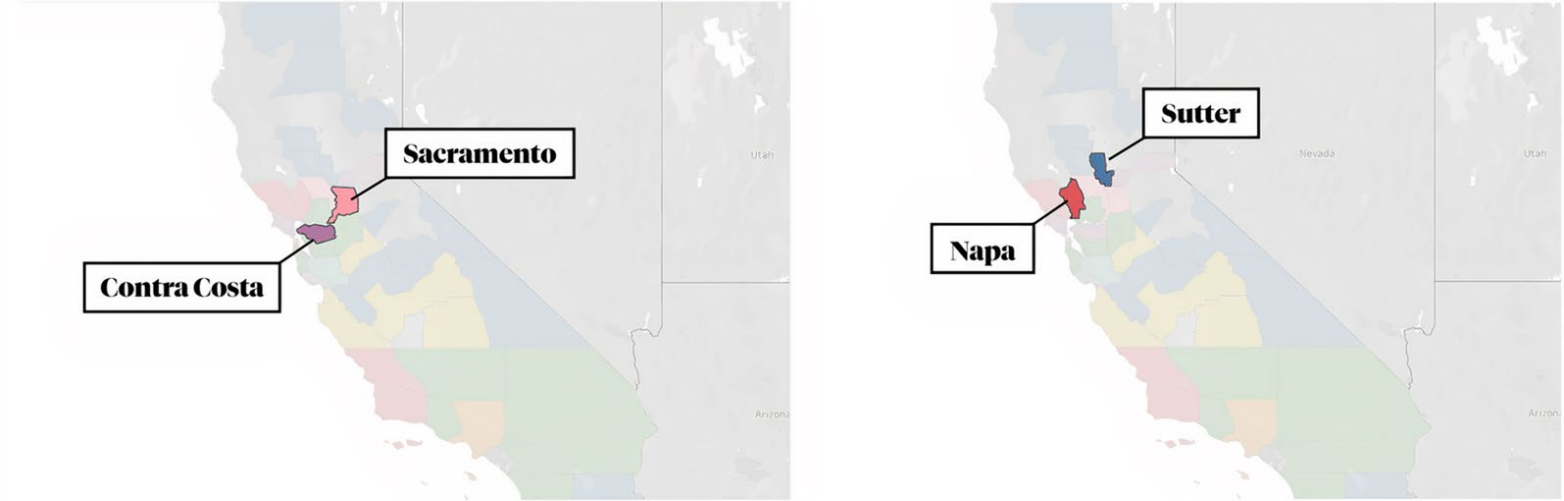
Geographic locations of Contra Costa and Sacramento (left) and Napa and Sutter (right)

The aim of this study was to investigate the impacts of the rollbacks rather than the impacts of the state shutdown. We assumed the shutdown applied to all counties equally, but the rollbacks would not, as each county specializes in a different economic sector that may or may not be regulated by EPA guidelines. Consequently, we identified an industry that would be impacted by the new EPA guidelines. From here we could then identify two counties: a treatment county, which had the industry, and a control county that was similar in every way to the treatment, except that it did not have the impacted industry (Fig. 1).

The fine print of the EPA guidelines suggests that the enforcement of pollution regulations is relaxed for all regulated entities, or economic sectors: “The consequences of the pandemic may constrain the ability of regulated entities to perform routine compliance monitoring, integrity testing, sampling, laboratory analysis, training, and reporting or certification [and]…may affect the ability of an operation to meet enforceable limitations on air emissions” [4]. Following the rollback of the enforcement of pollution regulations, the EPA released new guidelines that were designed with oil industries in mind [28] as the EPA advocated to “ease control on coal plants' toxic ash and loosen restrictions on mercury emissions” [38], establishing legislation that benefits fossil fuel industries. The agency established the new guidelines 3 days after executives from the American Petroleum Institute (API) petitioned the EPA to roll back “non-essential compliance obligations” [6]. Additionally, the EPA rollbacks meet the requests stated in the API petition, including (but not limited to) wet signature requirements, potential delay to project permits, deferred permit renewal applications, and potential to miss certifying laboratory equipment [30].

However, the manufacturing sector and oil industry are linked as the finished products of oil industries are the inputs for manufacturing sectors to convert the raw materials in finished goods [15]. The manufacturing sector is also an industry listed as being regulated by the EPA, indicating that manufacturing sectors could be impacted by the regulations.

**Fig. 2:**
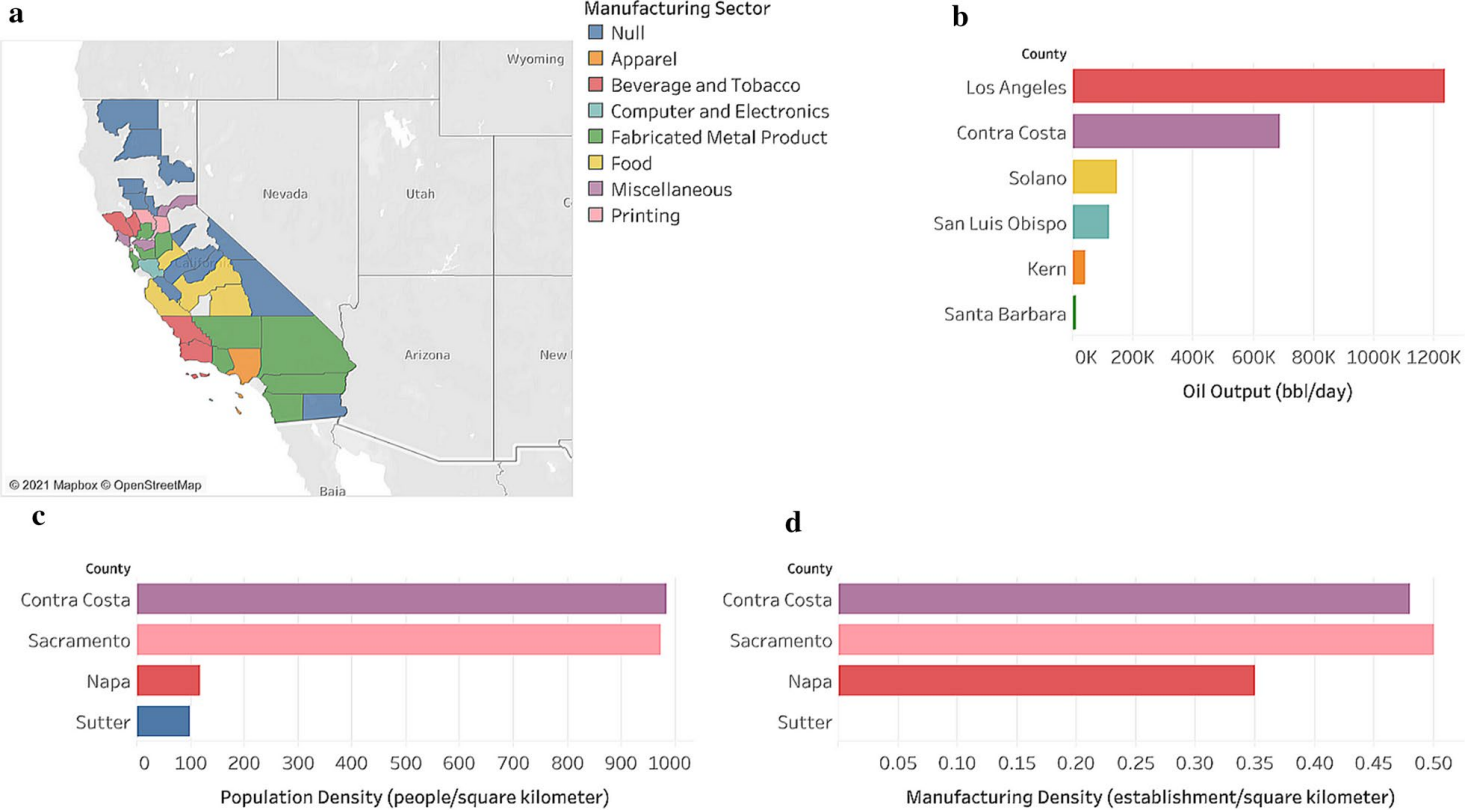
Economic overview of select California counties. Using data from the United States 2010 Census Bureau and the journal titled *Oil*, we graphed **a** main manufacturing sectors for 30 Californian counties, **b** output of major Californian oil refineries, **c** population density, and **d** manufacturing density

We created two pairs of treatment–control pairs: one for oil refineries and one for the manufacturing sector. Using oil refinery data [34], population data [7], and manufacturing establishment data [9], we identified two counties in California, Contra Costa and Sacramento, that have similar population densities, manufacturing establishments densities, and general climates. However, Contra Costa has a major oil industry as it has four oil refineries which produce a significant amount of oil each day (Fig. 2b), while Sacramento does not. We identified two other counties, Napa and Sutter, which have similar population densities and general climates. Napa, according to the 2010 United States Census Bureau, ranked in the top 30 Californian Manufacturing Cities, while Sutter did not, indicating that the manufacturing sector in Sutter appears to not be significant using industrial data from 2010.

### Statistical analysis

To determine association, we programmed in R to conduct t-tests. We split the data into a pre-shutdown and pre-rollback time series, which was composed of weekly and daily data before March 31, 2020, and a post-shutdown and post-rollback time series, which was composed of data after April 1, 2020. A two-sided paired t-test was used when comparing the treatment to control while a two-sided unpaired t-test was used when comparing pre-shutdown and pre-rollback to post-shutdown and post-rollback data.

## Results

### Measuring economic activity

**Fig. 3:**
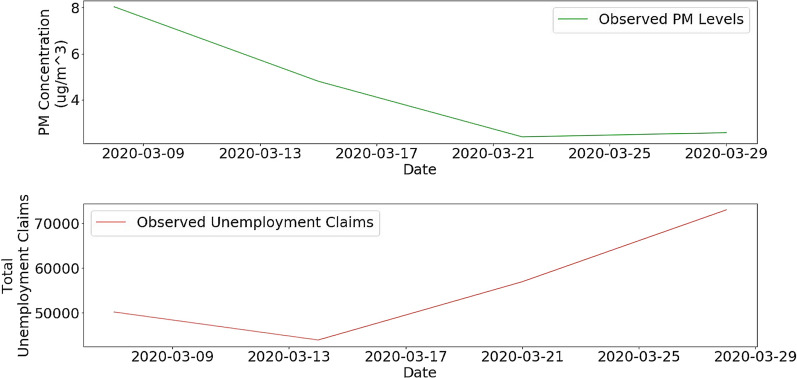
Comparison of weekly PM_2.5_ levels (top) and local unemployment levels (bottom) in Fresno, California in March 2020

Since GDP is recorded over 3 months and we wanted more frequent data, we sought an economic proxy that could act as a metric of economic activity concerning PM_2.5_ levels. After utilizing K-Nearest Neighbors Imputer to predict unemployment levels by county, we plotted weekly mean PM_2.5_ values against weekly unemployment values. The month of March experienced a significant change in unemployment and pollution with a 45% increase in unemployment and a 74% decrease in PM_2.5_ for the county of Fresno (Fig. 3). The results in Fig. 3 indicate an inverse relationship between unemployment and PM_2.5_ for the month of March.

The relationship can also be quantitatively established by running a correlation coefficient test on weekly pollution and unemployment data from February 16, 2020, to May 31, 2020. As shown in Table 1, the negative correlation coefficient values indicate that there is an inverse relationship between these two variables with some counties, such as Napa, Sacramento, and Sutter, exhibiting a stronger inverse relationship between unemployment and pollution than other counties, such as Contra Costa.

**Table 1:** Correlation coefficients showing the statistical relationship between weekly PM_2.5_ and local unemployment levels

County	Correlation coefficient
Contra Costa	− 0.4435
Napa	− 0.60851
Sacramento	− 0.66302
Sutter	− 0.68531

### Evaluating the economic viability of certain industries

**Fig. 4:**
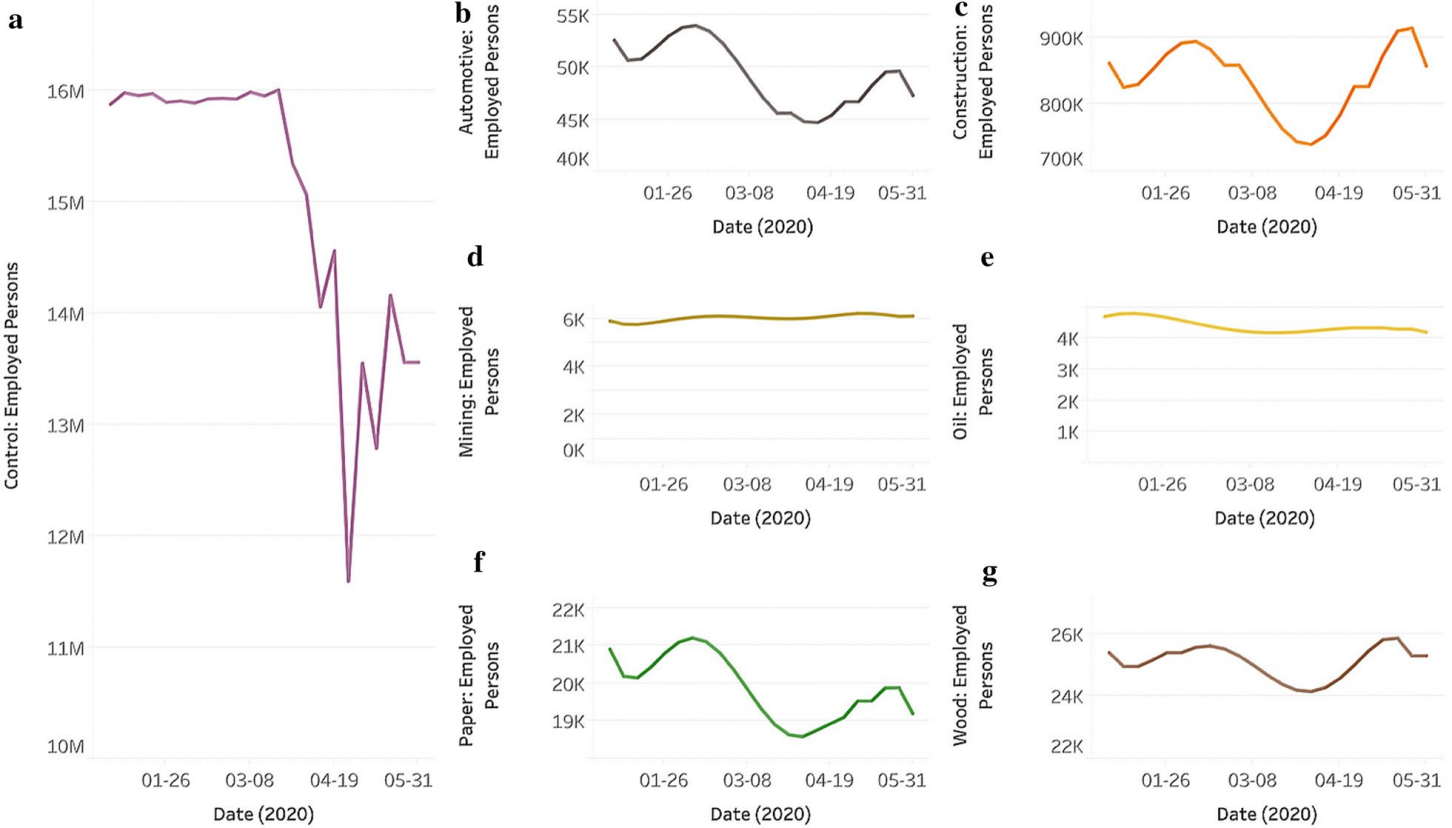
Total employed persons for treatment and control industries. Using an algorithm to predict an industry’s total employment, graphs for total employed persons for **a** all control, **b** automotive, **c** construction, **d** mining, **e** oil**, f** paper, and **g** wood industries were created

After affirming that unemployment could be a measure of economic activity on a county level, we evaluated the economic health of several state industries using employment data. We developed a model to predict total employed persons for specific industries weekly using monthly employment data provided by the California Employment Department [17], producing a richer data set. These values were then plotted in Fig. 4, comparing the employment values of treatment industries, or industries that could be affected by the rollbacks, to those of control industries, or industries that could be unaffected by rollbacks.

Data were then split into two categories: pre-shutdown and rollback, or weekly employment data that occurred before March 31, 2020, and post-shutdown and rollback, or weekly employment data that occurred after April 1, 2020. California issued a statewide stay-at-home order on March 19, 2020, and the EPA rolled out new guidelines on March 26, 2020.

Two two-sided paired t-tests, one using pre-shutdown and pre-rollback data and the other using post-shutdown and post-rollback data, were conducted using the employment values of a treatment industry and those of control industries. For these two t-tests, our null hypothesis stated that total employed persons for treatment and control industries were not statistically different before or after COVID and rollbacks. Our alternative hypothesis stated the opposite: the employment values for treatment and control industries were statistically significant before and after COVID and rollbacks. Both paired t-tests produced statistically significant values (*p* < 5 × 10^–11^) for each of the 6 treatment industries, possibly indicating that the employed persons of treatment industries are indeed different from those of control industries before and after the state shutdown and EPA rollback.

A two-sided unpaired t-test was conducted comparing pre-shutdown and pre-rollback employment data to post-shutdown and post-rollback data for the control industries and each of the six treatment industries. The null hypothesis stated that there was no statistically significant difference in an industry’s employed persons after shutdown and rollbacks, while the alternative hypothesis indicated that there was a statistically significant difference in an industry’s employment values after shutdown and rollbacks. As shown in Table 2, most industries, including the control industries, turned up statistically significant (*p* < 0.05). On the other hand, other industries, such as Construction and Wood Manufacturing, produced p-values that were not statistically significant (*p* > 0.05) (Table 2).

**Table 2:** P-values of employed persons before and after shutdown and rollbacks (two-sided unpaired t-test)

Industry	*p *value
Control	0.0001803
Automotive	0.0001606
Construction	0.319
Mining	0.003052
Oil	0.009941
Paper	0.0001068
Wood	0.4355

### Evaluating air pollution in treatment–control pairs

We collected daily PM_2.5_ data [19] from the oil refinery treatment–control pair (i.e., Contra Costa and Sacramento) and manufacturing treatment–control pair (i.e., Napa and Sutter). This data was averaged weekly and later split up into pre-shutdown and pre-rollback data, 2019 spring or early 2020 data, and post-shutdown and post-rollback data, data after April 1, 2020.

PM_2.5_ values before March 31, 2019, for a treatment and control county were used as pre-shutdown and pre-rollback data and inputted into a t-test. Weekly and daily data samples were used.

Weekly spring and summer PM_2.5_ levels from 2019 were used as pre-shutdown and pre-rollback data. Pre-shutdown and rollback data for a treatment county were compared to those of its control county in a two-sided paired t-test in R. The null hypothesis stated that the type of industry present in the treatment county was not associated with PM_2.5_ levels. The alternative hypothesis stated that the presence of the industry in the treatment county was associated with PM_2.5_ levels. As shown in Table 3, the treatment–control pair for oil industries produced statistically significant values using weekly and daily data (*p* < 0.05). On the other hand, the manufacturing treatment–control pair did not test statistically significant (*p* > 0.05) (Table 3).

**Table 3:** P-values of pre-shutdown and pre-rollback PM_2.5_ values for treatment–control counties (two-sided paired t-test)

Frequency	Treatment–control pair	
	Contra Costa–Sacramento	Napa–Sutter
Weekly	0.003271	0.7945
Daily	2.60E-07	0.621

Data from April 1, 2019, to June 30, 2019, were used as pre-shutdown and pre-rollback data. Data from April 1, 2020, to June 30, 2020, were used as post-shutdown and post-rollback data. Pre- and post-shutdown and rollback were compared for each county in a t-test. Daily and weekly data samples were used.

Weekly spring and summer PM_2.5_ data from 2019 and 2020 were used as pre- and post-shutdown and rollback data, respectively. Pre-shutdown and pre-rollback data were compared to post-shutdown and post-rollback data for each county in an unpaired two-sided t-test. The null hypothesis stated that a county’s PM_2.5_ levels did not change after the state shutdown and EPA rollbacks. The alternative hypothesis stated that PM_2.5_ levels did change after the state shutdown and EPA rollbacks. As shown in Table 4, Sacramento consistently turned up statistically significant (*p* < 0.05) while Contra Costa did not (*p* > 0.05).

**Table 4:** *p *values of pre- and post-shutdown and rollback PM_2.5_ levels (two-sided unpaired *t*-test)

Frequency	County			
	Contra Costa	Sacramento	Napa	Sutter
Weekly	0.3349	0.003492	0.1401	0.7499
Daily	0.1459	1.48E-08	0.01304	0.7815

Data from April 1, 2020, to June 30, 2020, were used as post-shutdown and post-rollback data for each treatment and control county and were inputted into a t-test. Daily and weekly samples were used.

Spring and summer PM_2.5_ data 2020 were used as post-shutdown and post-rollback data. Post-shutdown and post-rollback data for the treatment and control county were compared in a two-sided paired t-test. The null hypothesis stated that the PM_2.5_ levels from the treatment county were not statistically significant to those of its control county. The alternative hypothesis stated that the PM_2.5_ levels of the treatment county were statistically significant to those of its control county. As shown in Table 5, the oil refinery treatment–control pair produced statistically significant p-values (*p* < 0.05) using weekly and daily data (Table 5). However, the manufacturing treatment–control pair produced p-values that were not statistically significant (*p* > 0.05) (Table 5).

**Table 5:** *p *values of post-shutdown and post-rollback PM_2.5_ data for treatment–control pairs (two-sided paired *t*-test)

Frequency	Treatment–control pair	
	Contra Costa–Sacramento	Napa–Sutter
Weekly	6.17E-07	0.08321
Daily	2.20E-16	0.0009928

**Fig. 5:**
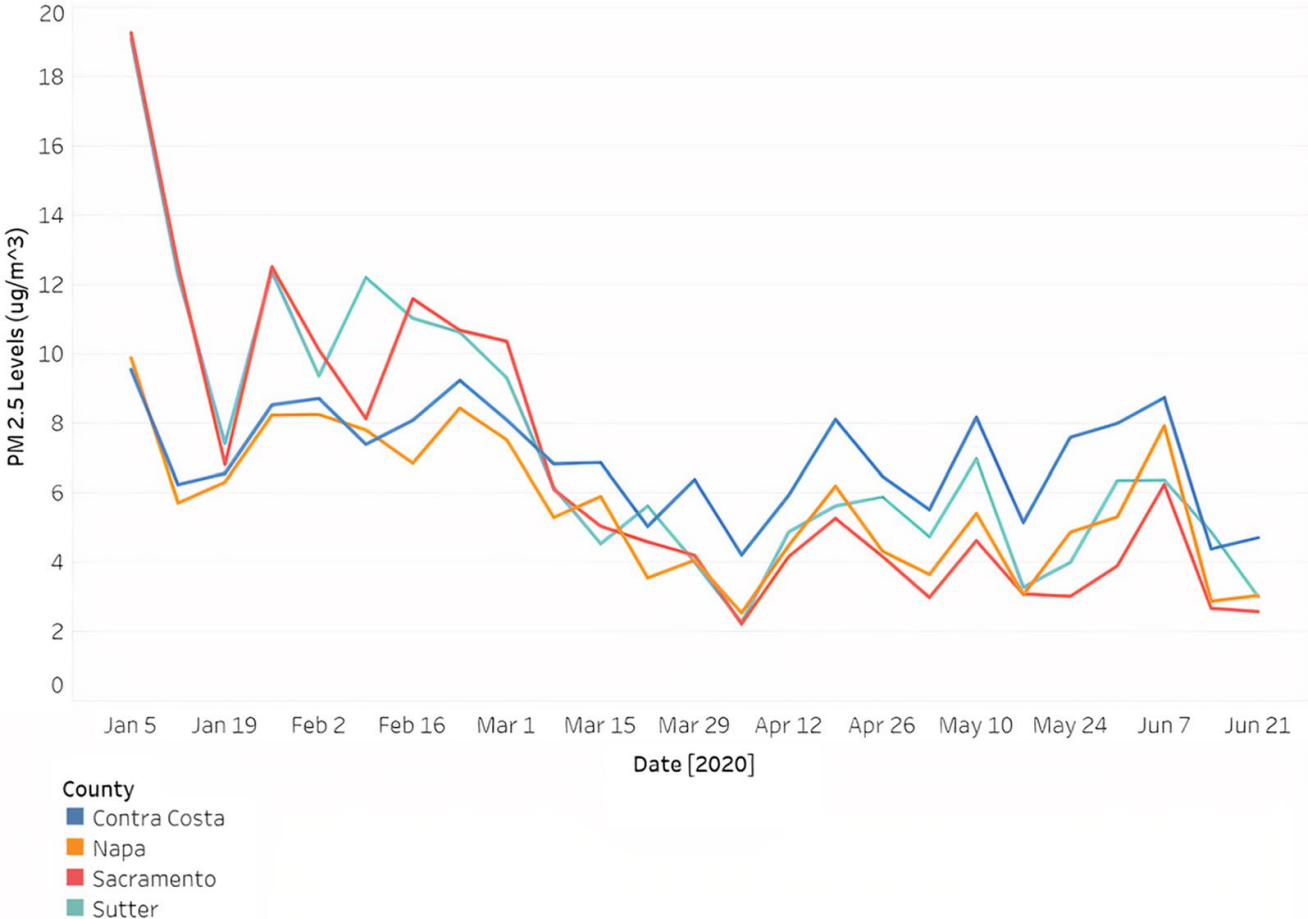
Weekly PM_2.5_ levels across all 4 counties from January 2020 to June 2020

In Fig. 5, we noticed that the control of the oil industry treatment–control pair (Sacramento) had higher PM_2.5_ levels than its treatment (Contra Costa) before the state shutdown and EPA rollbacks. However, we noticed that this behavior is switched after the state shutdown and EPA rollbacks: Contra Costa now has higher levels of PM_2.5_ than Sacramento. This behavior is not the same for the manufacturing treatment–control group. Sutter has higher PM_2.5_ levels than Napa, but this difference is not as consistent after shutdown and rollbacks.

From January 1, 2020, to June 30, 2020, a control county’s PM_2.5_ levels were subtracted from a treatment county’s PM_2.5_ levels. Data before March 31, 2020, became pre-shutdown and pre-rollback data while data after April 1, 2020, became post-shutdown and post-rollback data. For each treatment–control pair, pre- and post-shutdown and rollback data were inputted into a t-test.

To determine whether the differences in PM_2.5_ levels for the treatment and control county before and after shutdown and rollbacks were statistically significant, we subtracted the PM_2.5_ values of a control county from those of its treatment county from January 1, 2020, to June 30, 2020. Data from January 1, 2020, to March 31, 2020, acted as pre-shutdown and pre-rollback data, while data from April 1, 2020, to June 31, 2020, acted as post-shutdown and post-rollback data. These differences were then inputted into an unpaired two-sided *t*-test. The null hypothesis states that the difference between the treatment–control pair before and after the state shutdown and EPA rollbacks were not statistically significant, whereas the alternative hypothesis states that the differences after the state shutdown and EPA rollbacks were statistically significant. As shown in Table 6, the oil industry treatment–control pair has a higher statistically significant p-value (*p* < 0.0001) than the p-value for the manufacturing treatment–control pair.

**Table 6:** *p *values of PM_2.5_ differences before and after shutdown and rollbacks (two-sided paired *t*-test)

Treatment–control pair	*p *value
Contra Costa–Sacramento	0.0002155
Napa–Sutter	0.04157

## Discussion

Our study seeks to provide an objective analysis of the impacts EPA regulation rollbacks could have on the economic viability of certain industries and local air quality. A correlation coefficient test conducted on total unemployed persons and local PM_2.5_ levels revealed that total unemployment numbers and local air quality were inversely related (Table 1). We used employment as a metric for gauging the economic health of an industry and PM_2.5_ levels as a standard for measuring local air quality.

When determining whether the EPA’s enforcement rollbacks were bolstering the economic viability of certain industries, we found that the association between the EPA rollbacks and the economic health of a regulated industry varied significantly—some industries are financially worse off while others are better off after rollbacks. We classified employment data before March 31, 2020 as pre-shutdown and pre-rollback data. Data after April 1, 2020 were classified as post-shutdown and post-rollback data since rollbacks and shutdown occurred approximately at the same time. When pre- and post-shutdown and rollback data for each industry were inputted into a two-sided unpaired t-test, p-values for the control, automotive, mining, oil, and paper industries were statistically significant (*p* < 0.01) (Table 2). Because of the t-test conducted in Table 2, we can be confident that the employment values for these industries after the shutdown and rollbacks are not similar to those before the shutdown and rollbacks. For many of these industries, such as automotive, oil, and paper, current employment values have not returned to pre-shutdown and pre-rollback levels (Fig. 4). When comparing predicted employment values in May 2020 to recorded employment values in January 2020, the oil industry experienced a 12.5% decline in employment numbers. An August 2020 fossil fuel employment memorandum supports Fig. 4’s depiction of a downward state trend in oil employment numbers. According to the memorandum, California lost a total of 5,349 fossil fuel jobs between March 2020 to July 2020—a 14.7% decline [26]. On the national level, the fossil fuel industry lost 118,007 jobs between March 2020 to July 2020—a 15.5% decline in employment [26]. Additionally, this report indicated that the oil sector lost the most workers in the fossil fuel industry, with most oil job losses occurring in the extraction activities department [26]. This decline can be attributed to the downward trend in energy demand as Americans chose to stay at home and out of their cars [26]. Even with the suspension of enforcement regulations, fossil fuel companies still furloughed or laid off their workers [26]. Our results and the memorandum’s findings indicate that the agency’s suspension of enforcement regulations is not correlated with the economic health of the oil industry in the COVID-19 economic crisis. Additionally, the memorandum lends further credence to our study. Since California and the United States experienced similar percentage declines in fossil fuel employment, California acts as an effective proxy for nationwide trends from March 2020 to July 2020.

On the other hand, the mining industry’s employment values have returned and surpassed pre-shutdown and pre-rollback levels, indicating that its economic viability is currently better than what it had been at the beginning of the year. On the other hand, other industries, such as the construction and wood industries, produced p-values that were not statistically significant (*p* > 0.05) (Table 2). These statistically not significant p-values could be associated with the workforce stability of these industries. For instance, industries like construction tend to employ temporary workers which made up 15.5% of employees in the construction industry in 2014 [10]. Doing so made it easier for companies to quickly adjust their labor [10], potentially making it easier to quickly rebuild during a state shutdown.

To create a richer data set, we predicted employment values on a weekly basis for regulated industries. A polynomial regression algorithm fitted a quartic function to each of the treatment industry's employment values. Any other type of function would result in inaccurate predictions. While Table 7 indicates that the polynomial regression algorithm could accurately predict data, additional data would allow the algorithm to identify stronger correlations with the data and thus improve the accuracy of the algorithm.

**Table 7:** Polynomial regression algorithm accuracies for the six treatment industries

Industry	Model accuracy (%)
Automotive	98.521
Construction	90.226
Mining	93.399
Oil	99.645
Paper manufacturing	97.526
Wood manufacturing	89.573

Generally speaking, the results from Fig. 4 and Table 2 indicate that most industries are doing worse following the shutdown and rollbacks. A qualitative analysis of Fig. 4 indicates that the rollbacks might not be associated with increased economic viability for all industries, even for the oil industry which championed the rollbacks. To quantitatively ascertain this assertion, a paired t-test could be conducted to look into the differences between the employment levels of a treatment industry and those of the control industries. The null hypothesis for this procedure must take into account the differences between treatment and control under normal circumstances. The null hypothesis would be different for each industry and would have to be extrapolated from the average difference in performance between each one of the industries and the treatment group.

Our study also sought to answer an environmental question: were these rollbacks upholding the agency’s mission of protecting the environment, or more specifically, not negatively impacting the environment? To answer this question, the focus of the study shifted from industry-based to location-based.

To determine whether certain industries were associated with PM_2.5_ levels, we compared 2019 spring and summer data for treatment–control pairs in a two-sided paired t-test and obtained statistically significant p-values for the Contra Costa–Sacramento pair (*p* < 0.005) but not for the Napa–Sutter pair (*p* > 0.05) (Table 3), indicating that the presence of an oil refinery could be associated with PM_2.5_ levels. This finding is supported by a study conducted by Zhang et al. who found that excluding meteorological factors, the production of natural gas, industrial boilers, ore, tractors, nuclear power, and locomotives have the highest association with PM_2.5_ concentration in China [43]. Zhang et al.’s report also suggests that the presence of a manufacturing sector could be associated with PM_2.5_ levels. Using data from DataUSA [12], Table 8 indicates that Napa and Sutter’s economies were not as well matched as we initially thought, thus producing the statistically not significant p-values (*p* > 0.05) in Table 3. Consequently, we cannot associate the presence of a manufacturing section with PM_2.5_ levels.

**Table 8:** Overview of concentration of occupations in select Californian counties

Occupation	Contra Costa (%)	Sacramento (%)	Napa (%)	Sutter (%)
Agriculture, forestry, fishing, hunting	0.50	0.60	6.40	5.20
Mining, quarrying, oil and gas extraction	0.30	0.00	0.30	2.00
Construction	6.90	5.70	5.60	13.30
Manufacturing	6.90	4.90	11.70	10.00
Wholesale trade	2.40	2.50	2.90	1.00
Retail trade	10.80	10.40	11.10	9.70
Transportation and warehousing	3.80	3.80	1.90	2.00
Utilities	1.10	1.00	0.60	3.00

To determine whether PM_2.5_ levels were significantly different after the shutdown and rollbacks, spring 2019 data were compared to spring 2020 daily data for each county, producing p-values that were statistically significant for Sacramento (*p* < 0.01) (Table 4). This result potentially indicates that the PM_2.5_ levels after the shutdown and rollbacks were different from those before due to the state lockdown. P-values for Contra Costa and Sutter were not statistically significant (*p* > 0.05) (Table 4), indicating that PM_2.5_ values after the shutdown and rollbacks were not statistically different than those before. Contra Costa has a significant oil industry while its control county does not. These oil refineries could be taking advantage of the present EPA regulations, causing the same amount of pollution levels to be produced after the shutdown and rollbacks. Napa tested inconclusive; the use of a weekly sample did not produce a statistically significant *p*-value (*p* > 0.05), whereas a daily sample did (*p* < 0.05) (Table 4). A daily data sample might be richer and noisier than a weekly data sample, causing this variation.

To determine whether a treatment and its control county’s PM_2.5_ levels were statistically different following the state shutdown and EPA rollbacks, spring 2020 data for each of the treatment–control pairs were inputted into a two-sided paired* t*-test. The Napa–Sutter pair tested inconclusive as weekly and daily data samples produced different p-values. The Contra Costa–Sacramento pair produced a statistically significant p-value (*p* < 1.0 × 10^–6^) (Table 5). While Contra Costa and Sacramento had similar economies, Contra Costa has a higher concentration of fossil fuel industries than Sacramento (Table 8). These oil refineries in Contra Costa could have remained in operation during the pandemic and continued emitting PM_2.5_, causing the statistical difference in pollution levels between Contra Costa and its control county.

To investigate the relationships seen in Fig. 5, the differences in pollution between the treatment–control pairs before and after the lockdown and rollbacks were compared in a two-sided unpaired t-test. Both treatment–control pairs tested statistically significant (*p* < 0.05) (Table 6). Since all four counties were influenced by COVID-19, but experienced varying degrees of impact from rollbacks as a result of their economies, certain industries, particularly oil refineries, could take advantage of the EPA suspension to continue producing the same amount of pollution well after the state lockdown and rollbacks.

**Fig. 6:**
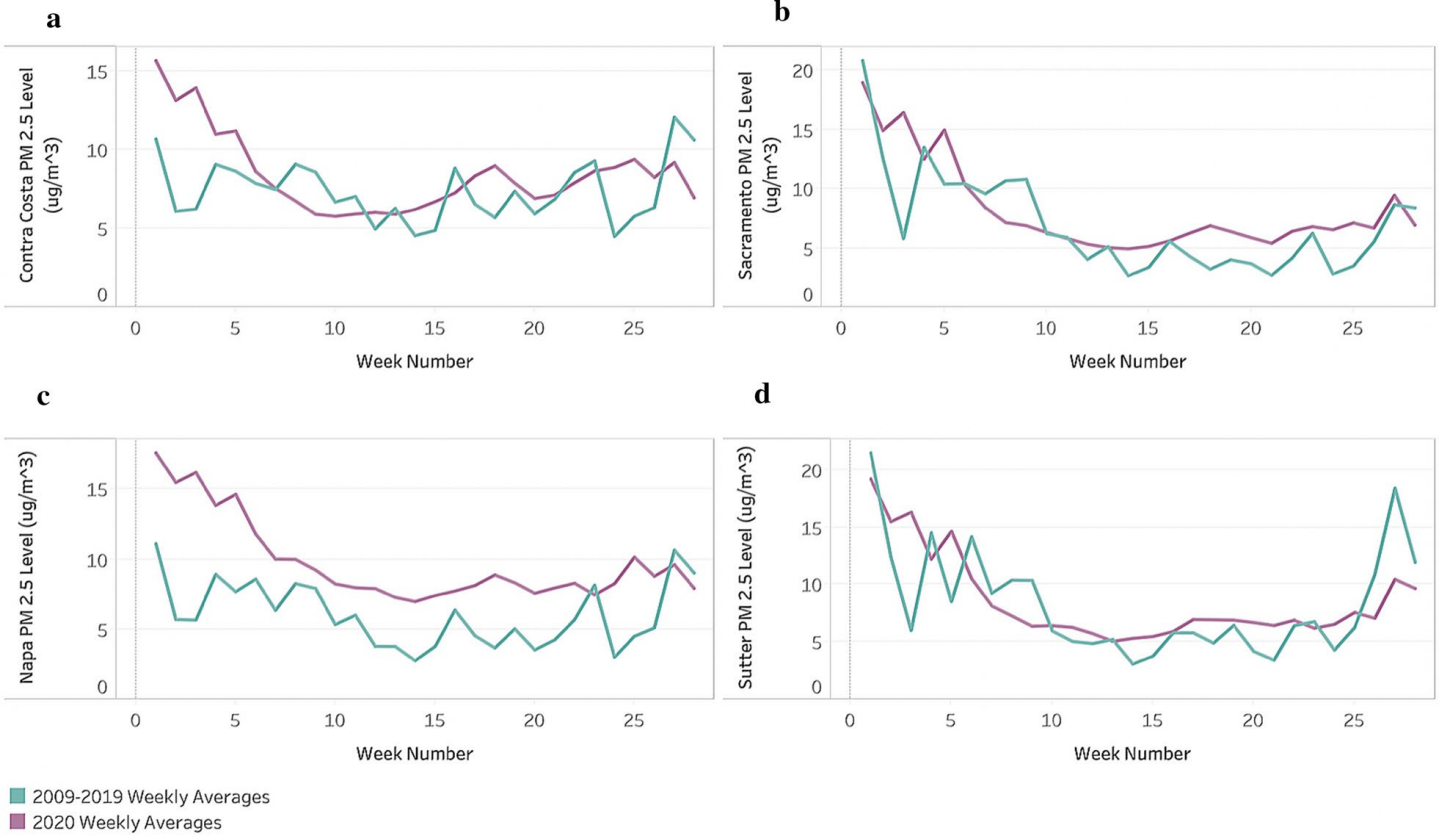
Difference between average 2009–2019 PM_2.5_ levels and 2020 PM_2.5_ levels for **a** Contra Costa, **b** Sacramento, **c** Napa, and **d** Sutter

The results produced by the t-tests are not due to seasonality as spring and summer 2019 data were compared to spring and summer 2020 data. The behavior of PM_2.5_ levels in 2020 do not always mirror historical pollution data. In Fig. 6, some counties, such as Napa, continued to have higher pollution levels in 2020 than in the past decade, despite California’s state lockdown.

While we tried to eliminate as many hidden variables as possible by making the treatment and control counties similar to one another, our methodology and results could be improved. For instance, Contra Costa and Sacramento might experience the same general climate, but have varying microclimates [37], which could then influence pollution levels [33]. While Table 8 validates our conclusion that Contra Costa has an oil industry and Sacramento lacks one, Napa and Sutter devoted differing amounts of their population to fossil fuel industries and construction, industries that are well known for exacerbating pollution levels [14]. According to Table 8, Sutter’s manufacturing sector is almost equal to that of Napa’s, meaning that its manufacturing sector was not as insignificant as we initially thought.

We can conclude that Contra Costa and Sacramento are a better match than Napa and Sutter. Based on Table 4, we can conclude that oil industries could be producing the same amount of pollution levels before and after the state shutdown, potentially as a result of these regulations. While we do not have sufficient evidence to say the same for manufacturing sectors, it is important to note the environmental repercussions of what could happen when the state lockdowns are lifted and industries, such as oil refineries, can return to normal levels of activity without the presence of regulations to limit emissions.

Another study that investigated the EPA’s suspension of enforcement regulations came to similar conclusions. Following the agency’s rollback of enforcement, counties with more Toxic Release Inventory (TRI) sites saw increases in pollution while counties with fewer TRI sites saw a smaller increase in pollution [35].

While the EPA’s regulation suspension was lifted in August 2020, we advise against the establishment of similar legislation. The regulatory suspension in America was not an isolated incident as many countries around the world have deregulated environmental policies. Seeing that these guidelines fail to meet the economic expectations of oil and manufacturing executives and endanger environmental health, we ask policymakers to maintain policies that facilitate the planning, implementing, enforcing or monitoring of pollution in a pandemic. This recommendation is supported by a Delhi case study conducted by India’s National Air Program. After studying agricultural burning—a major source of air pollution in Delhi—and witnessing the significant decrease in primary pollutants but only a moderate decrease in PM 2.5, researchers recommended the execution of a comprehensive program for the prevention, control, and abatement of pollution [23]. Examples include developing emission baselines, mandating preparations and maintenance of pollution databases, establishing protocols and laboratories for continuous measurements, and building monthly and annual emission inventories [23]. Prior to the suspension, the EPA’s enforcement guidelines met some, if not most, of these recommendations. However, the agency’s suspension of these enforcement regulations and the international deregulation of environmental policies fly in the face of the Delhi case study’s recommendations. Rather than suspend pollution monitoring in a pandemic, we ask policymakers to uphold such policies as the suspension of them endangers environmental health in a pandemic. Policy changes should be carefully designed to achieve long-term goals. Short-term policy changes are ineffective and counter-productive.

## Conclusions

The statistical analysis of employment levels does not indicate a clear correlation between the EPA rollback regulations and the improved economic viability of certain industries during the COVID-19 pandemic. Additionally, a statistical analysis of PM_2.5_ levels indicates that certain counties experienced significant changes in pollution levels after state lockdowns and rollbacks while other counties containing certain pollution-associated industries, namely oil refineries, are experiencing pollution levels similar to those of 2019.

This study not only notes California’s below-average levels of economic activity, but also emphasizes the environmental repercussions of what might result from the suspension of enforcement regulations. If pollution emissions are not checked with the enforcement of regulations, residents face the risk of a polluted environment during the COVID-19 pandemic.

Most EPA-regulated sectors have lower employment values during COVID-19 than before the pandemic, indicating that these sectors are economically worse off. In a time when financially aiding large and small businesses is considered a paramount priority, these guidelines fail to satisfy the expectation oil and manufacturing executives have proposed: additional financial assistance in a time of economic recession. Industries that have backed these regulations continue to face declining economic health as seen in decreasing employment levels (Additional files 1, 2, 3, 4, 5, 6, 7, 8, 9, 10, 11, 12, and 13).

This study’s statistical analysis of employment levels by occupation and PM_2.5_ levels by county provides the EPA’s specialists, United States’ legislators, and international policymakers with the needed information for designing effective pollution and industry regulations. This study highlights the need for the reconsideration of similar regulations. Further research could reinforce the environmental and economic findings of this study (Additional files 14, 15, 16, 17, 18, 19, 20, 21, 22, 23, 24, 25, and 26).

## Supplementary Information

**Additional file 1.** California.csv

**Additional file 2.** California2009.csv

**Additional file 3.** California2010.csv

**Additional file 4.** California2011.csv

**Additional file 5.** California2012.csv

**Additional file 6.** California2013.csv

**Additional file 7.** California2014.csv

**Additional file 8.** California2015.csv

**Additional file 9.** California2016.csv

**Additional file 10.** California2017.csv

**Additional file 11.** California2018.csv

**Additional file 12.** California2019.csv

**Additional file 13.** CaliforniaEmploymentIndustry.csv

**Additional file 14.** CaliforniaEmploymentIndustry_Control.csv

**Additional file 15.** CaliforniaUnemployment.csv

**Additional file 16.** ContraSacraNapaSutt2009-2020

**Additional file 17.** CorrelValues.csv

**Additional file 18.** Jan-Jun-Updated.csv

**Additional file 19**. RawFresnoUnemployment.csv

**Additional file 20.** ResearchIntoCaliforniaVer2.csv

**Additional file 21.** updatedAutoData.csv

**Additional file 22.** updatedConstructionData.csv

**Additional file 23.** updatedMiningData.csv

**Additional file 24.** updatedOilData.csv

**Additional file 25.** updatedPaperData.csv

**Additional file 26.** updatedWoodManufacturing.csv

## Data Availability

The dataset supporting the conclusions of this article is available in the Environmental Protection Agency’s Outdoor Air Quality Database, https://www.epa.gov/outdoor-air-quality-data/download-daily-data. Other datasets supporting the conclusions of this article are included within the article and its additional files.

## Abbreviations

API: American Petroleum Institute
AQI: Air Quality Index
BRR: Bayesian ridge regression
COVID-19: Coronavirus disease 2019
EPA: Environmental Protection Agency
GDP: Gross Domestic Product
GSP: Gross State Product
KNN: K-Nearest Neighbors
PM_2.5_: Fine Particulate Matter 2.5
NaN: Not a Number
TRI: Toxic Release Inventory
WHO: World Health Organization
